# Personalized Biomechanical Analysis of the Mandible Teeth Behavior in the Treatment of Masticatory Muscles Parafunction

**DOI:** 10.3390/jfb12020023

**Published:** 2021-04-09

**Authors:** Denis Gribov, Mikhail Antonik, Denis Butkov, Alexandr Stepanov, Pavel Antonik, Yaser Kharakh, Anton Pivovarov, Sergey Arutyunov

**Affiliations:** 1 Department of Applied Mechanics, Bauman Moscow State Technical University, 105005 Moscow, Russia; gribov_denis@mail.ru; 2 Department of Propaedeutics of Dental Diseases, A.I. Yevdokimov Moscow State University of Medicine and Dentistry, 127473 Moscow, Russia; michail@antonikm.ru (M.A.); denis.stomservice@mail.ru (D.B.); stepanovmd@list.ru (A.S.); pasha-oop@yandex.ru (P.A.); anton_pivovarov@bk.ru (A.P.); sd.arutyunov@mail.ru (S.A.)

**Keywords:** bruxism, case reports, dental occlusion, balanced, dental occlusion, dental stress analysis, finite element analysis, occlusal splints, polymethyl methacrylate, printing, three-dimensional technology, dental

## Abstract

A 3D finite element model of the mandible dentition was developed, including 14 teeth, a periodontal ligament (PDL), and a splint made of polymethylmethacrylate (PMMA). The study considered three design options: 1—the case of splint absence; 2—the case of the splint presence installed after manufacture; and 3—the case of splint presence installed after correction (grinding) performed to ensure a uniform distribution of occlusal force between the teeth. For cases of absence and presence of splint, three measurements of the functional load were performed using the T-Scan III software and hardware complex (TekScan, Boston, MA, USA). It was found that the presence of a splint led to a decrease in the total value of the occlusive load and to a uniform distribution between all the mandible teeth. The occlusal force was considered as a static vertical force evenly distributed between the nodes belonging to the occlusive surface of the corresponding tooth for the first design option and the occlusal surface of the splint for the second and third ones, respectively. As a result of the study, it was concluded that the splint usage was effective in order to change the distribution of the functional load during the treatment of proved masticatory muscles’ parafunction; the safety of using a splint for teeth and surrounding tissues under the influence of the considered functional load was shown; the potential applicability of PMMA as a structural material of a splint that had been used for the treatment of masticatory muscles’ parafunction was established.

## 1. Introduction

The movement of the mandible is based on the stretching reflex—the myotonic reflex. The monosynaptic stretching reflex actively ensures the mandible resting position along with reflexes that include temporomandibular joint (TMJ) receptors [1,2]. As applicable, the reflex transformation is carried out by adaptation mechanisms. In cases of occlusal obstacles, the trajectory of mandible movements may change, and in order to carry out chewing and speech functions, there is a need for a different muscle work scheme. Violation of the myotonic reflex can also be expressed as asynchronous muscle activity and an extreme increase in muscle activity—parafunction (nocturnal and diurnal bruxism). Parafunction of the mastication muscles is manifested in the form of involuntary and poorly controlled by patients contractions of the mastication and facial muscles: biting the lips, cheeks, tongue, clenching the teeth, and moving the mandible forward or to the side. More often, there are combinations of various parafunctions, which are less often due to neuroemotional stress and excessive physical exertion—hereditary predisposition or incorrect speech articulation and other reasons. Such violations lead to abrasion of teeth, pain in the mastication muscles, joint degeneration, and displacement of the articular disc and tooth [3,4,5].

According to various studies, the prevalence of bruxism reaches 90% [6,7,8]; moreover, the course of the disease aggravates with age [9,10,11].

Now, hypertonicity and parafunction of the mastication muscles (bruxism) are mainly of central origin [12,13]. Bruxism leads to occlusion disorders or pathologically asynchronous movements of the mandible head and disc, to functional overload of the TMJ [14,15]. Bruxism is defined as a sleep disorder and at the same time can be considered as a protective response of the body to stress; the release of adrenaline can stimulate the breakdown of glycogen in contracting skeletal muscles [16,17]. The result of such muscle activity is increased wear of the teeth hard tissues; however, the internal organs do not suffer from the “adrenaline attack” [18,19].

Bruxism causes prolonged nonfunctional sliding movements of the mandible relative to the maxilla with closed dentition, and therefore, the correction of occlusion does not contribute to the elimination of bruxism [20,21]. For the treatment of patients suffering from couple functions, correction of the neuropathic state is required, as well as the elimination of the causes of an unfavorable emotional background. Deprogramming and relaxation of the mastication muscles is possible in the case of using relaxing occlusal splints for a long period (3–4 weeks and longer); such devices are highly effective (relaxation splint, as well as splint increasing the vertical height) [22,23,24,25]. An occlusal splint is effective only when the neuromuscular system allows the presence of the splint in the oral cavity and occlusal changes that are caused by an occlusal splint [26]. Therefore, it is necessary to provide the proper correction of the occlusal splint before using it. The first correction of splint before usage is the most important because the earliest effect of muscle relaxation depends on it.

The aim of this work is to assess the effectiveness of a selected treatment option for mastication muscle parafunction by analyzing the stress–strain state (SSS) of individual biomechanical models of the mandible dentition for three cases: the case of splint absence; the case of splint presence installed after manufacturing; and the case of splint presence installed after correction (grinding), performed to ensure a uniform distribution of occlusal force between the teeth.

## 2. Materials and Methods

### 2.1. Ethical Aspects and Study Design

The study was conducted according to the guidelines of the Declaration of Helsinki and approved by the interuniversity Ethics Committee of A.I. Evdokimov Moscow State University of Medicine and Dentistry (protocol NO. 08–20). Informed consent was obtained from all subjects involved in the study. The study design was built in accordance with the level 2 of TOP guidelines.

### 2.2. Finite Element Model

As the study’s initial data, we considered a computed tomography (CT) of a patient (24 years old, female). The patient reported that she suffered from nocturnal bruxism. At the time of the study, dental treatment was not received. Previously prosthodontic and orthodontic treatment had not been performed. The patient had complaints of the masticatory muscles’ stiffness. The intraoral examination revealed a dystopia of the teeth, crowding of teeth in the anterior part of the dentition, well-defined severe attritional wear facets (with extending into dentin) located on molars, canines of the maxilla, and mandible (Figures S2–S4).

CT consisted of 550 DICOM images with voxel size 0.20 mm × 0.20 mm × 0.20 mm. Based on the data obtained, a 3-dimensional finite-element model of the mandible dentition was created, directly including 14 teeth with periodontal ligament (PDL).

**Figure 1:**
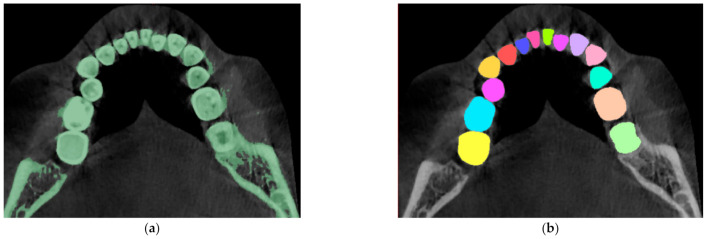
The result of one segmentation of the computed tomography (CT) scans: (**a**) automatic segmentation using the 1000–3000 Hounsfield units (HU) range; (**b**) the result of interactive image editing (selection of areas related to the teeth of the mandible, on the right).

The transformation of CT raster images into a three-dimensional solid model of the dentition was carried out using the Mimics 17.0 and 3-matic 6.1 software packages (Materialise, Leuven, Belgium). At the first stage, automatic segmentation of CT scans was performed, selecting an area related to the teeth by means of setting the appropriate range of X-ray density on the Hounsfield scale. When processing computed tomography images, the range from 150 to 1800 Hounsfield units (HU) was generally accepted for identifying bone tissue (compact and cancellous), and the X-ray density of tooth enamel reached 3000 HU [27]. However, on CT scans, areas belonging to both bone and surrounding soft tissues fell into the range of 150–3000 HU. Therefore, automatic segmentation was performed with the lower limit of the range increased to 1000 HU, and the results were corrected using interactive editing tools—the areas related to 14 teeth were separately highlighted (Figure 1).

**Figure 2:**
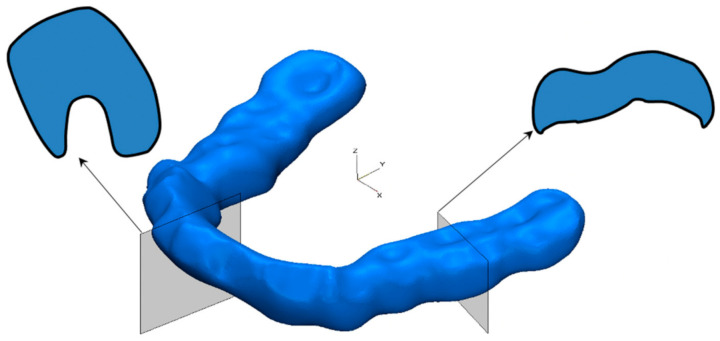
3D STL model of the splint showing the shape of the cross-sections in the areas of the central incisors and molars.

Further, based on the segmentation results, surface tooth models’ automatic creation was carried out with their subsequent smoothing and optimization of the triangular faces quality. PDL was modeled by generating additional surfaces equidistant from the root parts of the teeth by 0.25 mm. The option of installing a removable personalized splint on the entire mandible’s dentition of the mandible made by 3D printing was considered. The splint was modeled separately based on the results of processing the existing CT and intraoral scans of the dentition obtained with the 3Shape TRIOS 3 apparatus (3Shape, Copenhagen, Denmark) in the specialized software complex Zirkonzahn.Modellier (Zirkonzahn Gmbh, Gais, Switzerland), taking into account the absence of a gap between the inner surface of the splint and the teeth. The resulting virtual model of the splint (in *.STL format) was exported to the 3-matic 6.1 software package, where it was combined with the created model of the mandibular dentition (Figure 2).

After checking and adjusting the quality of all surfaces as required, a volumetric mesh of model components was created based on four nodal tetrahedral. Approximating the model components, the maximum length of the tetrahedron side was the following values: tooth—0.70 mm, periodontium—0.25 mm, and splint—0.50 mm. With the above parameters, the number of nodes for the model was 167,306, for elements—756,844.

The material of all components of the model was modeled as isotropic homogeneous linear elastic. For the splint material, the values of polymethylmethacrylate (PMMA) are specified [28,29]. The values of the mechanical characteristics of the model materials are given in Table 1.

After the completion of the model creation, it was exported to the ANSYS finite element complex (Ansys Inc., Canonsburg, PA, USA). Since all the components of the model consisted of four nodal tetrahedrons, they were assigned an element of the SOLID285 type with three translational degrees of freedom at each node. The fixation of the splint on the dentition was modeled by creating a fixed contact (bonded type) between the adjacent nodes belonging to the outer surface of the dental crowns and the inner surface of the splint.

### 2.3. Design Options and Loading Conditions

The main task of the study was to determine the values of design parameters for the components of the model upon the action of the functional load. The magnitude and distribution of functional load were determined using the T-Scan III software and hardware complex (TekScan, Boston, MA, USA), which was used to analyze the relative occlusal recorded by intraoral sensor and redistribution of forces at each moment in time graphically expressed by the symbol of a rhombus leaving behind a trajectory of movement (Figure S5). For each measurement, we performed three consecutive bites (“multibite scanning technique”). The maximum values that were determined using the automatic software function “maximum bite” were used for further calculations.

For this purpose, using the available virtual STL model, one experimental splint sample from PMMA Harz Labs (Harz Labs, Moscow, Russia) was made by 3D printing using an Asiga Max UV 3D printer (Asiga, Alexandria, Australia).

**Figure 3:**
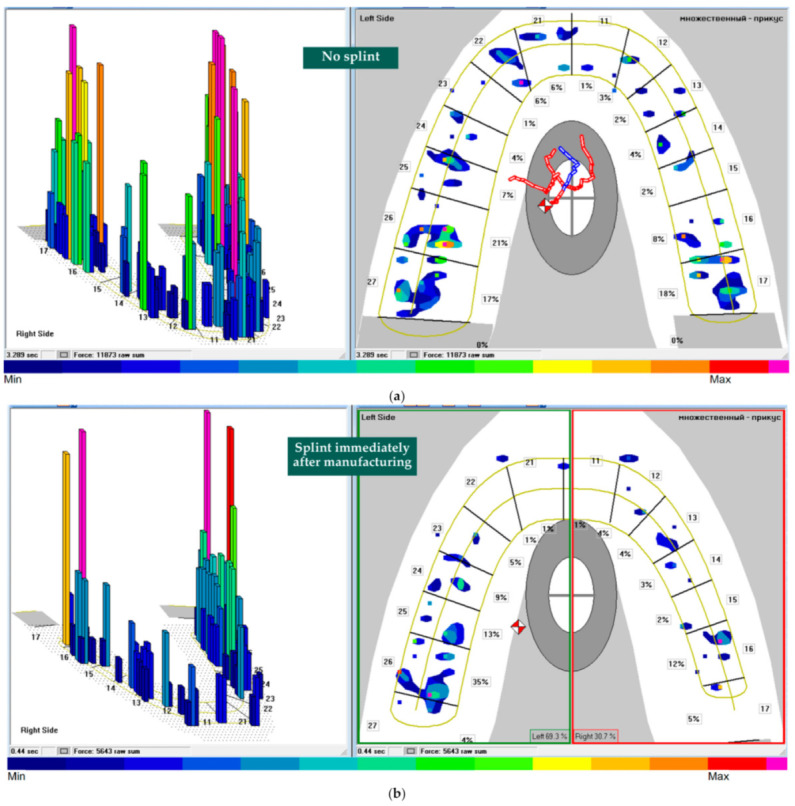

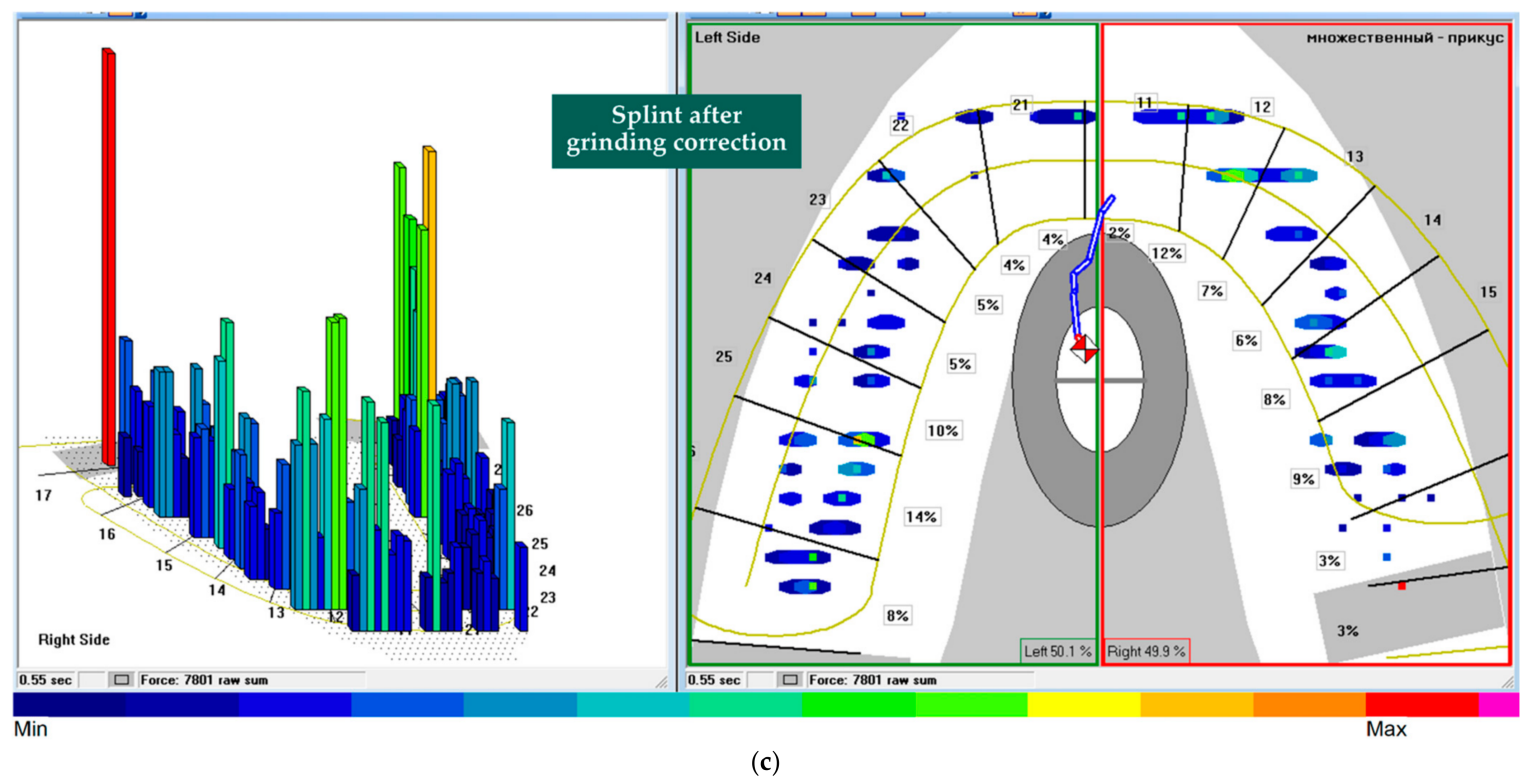
Results of the relative and percentage distribution of occlusal force between the teeth of the antagonists in the T-Scan III system software: (**a**) measurement without splint; (**b**) measurement with a splint, immediately after manufacture; and (**c**) measurement with a splint, after correction (grinding) was performed to ensure a uniform distribution of occlusal force between the teeth.

The initial measurement of functional loading (Measurement 1) using the T-Scan III was performed without a splint on the mandibular arch (Figure 3a), which determined that 61.5% of the total load was on the left side of the mandible and 64.0% on the molars 36, 37, 46, and 47 (tooth designations are given in accordance with two-digit FDI World Dental Federation notations (Geneva, Switzerland)). The measurement results (Measurement 2) shown in Figure 3b were obtained with the splint installed immediately after manufacture. Here, the left side of the mandible accounted for 69.3% of the total load and 35.0% for molar 36. In order to guarantee a more balanced distribution of occlusal force between the teeth, the initially manufactured splint was grounded (abraded) in local areas. The results of measurement (Measurement 3) that were obtained after the operation are presented in Figure 3c. The total occlusal load between the left and right sides of the jaw was distributed as 50.1%/49.9%, and 37.0% fell on molars 36, 37, 46, and 47. During measurements, the sensor sensitivity settings were set to “by default”.

In Figure 3, it can be seen that in all measurements the relative occlusal force (raw sum) is distributed between all teeth and is as follows: at measurement 1—11873, at measurement 2—5643, and at measurement 3—7801. The T-Scan III system does not allow one to determine the absolute value of the occlusal force. To convert the relative force in absolute (Newton (N)) force, the following linear equation (Equation (1)) [32] was used:

(1) $$F=0.021\times \left(raw\;sum\right)+8.022$$

The results of determining the absolute values of occlusal forces acting on perceived (antagonizing) teeth were as follows: for measurement 1—257.4 N, for measurement 2—126.6 N, for measurement 3—171.8 N, and their distribution is presented in Table 2.

Therefore, this study examined the calculated three options:

- Option 1: The model of the dentition without the installed splint;
- Option 2: The model of the dentition with a splint installed after production;
- Option 3: The model of the dentition with a splint installed after grinding.

**Figure 4:**
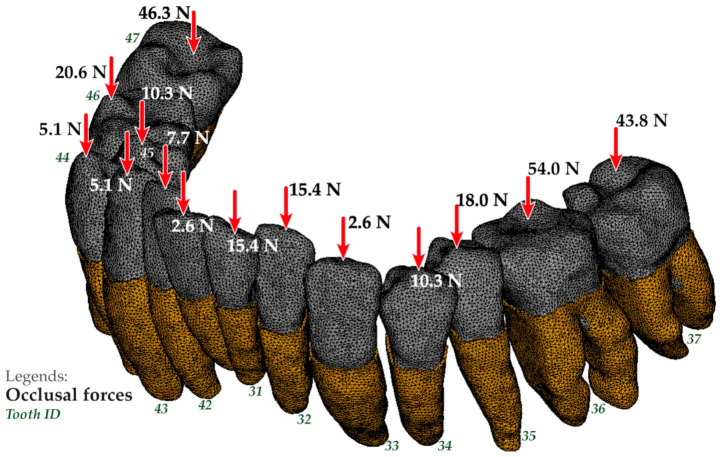
Finite-element model of the mandible dentition with indication of the occlusal forces application areas and the conditions for fixing the model for option 1.

**Figure 5:**
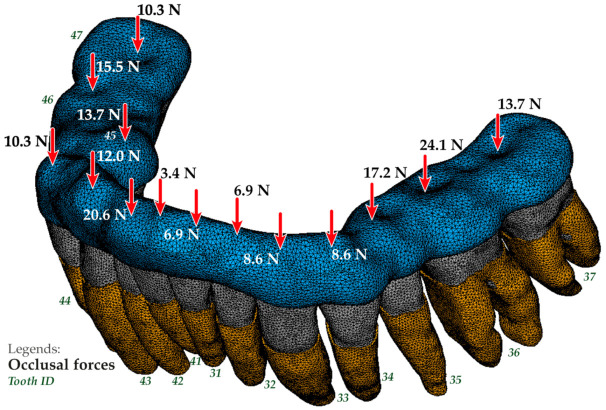
The finite-element model of the mandible dentition with indication of the occlusal forces application areas for option 3.

The finite element model of the mandible dentition with an indication of the occlusal forces application areas for design options 1 and 3 is shown in Figure 4 and Figure 5. The occlusal force was considered as a static vertical force evenly distributed between the nodes belonging to the crown occlusal surface of the corresponding tooth for the first design option and the occlusal surface of the splint for the second and third design options, respectively. The fixation of the model was carried out by fixing all degrees of freedom in the nodes belonging to the outer surface of the periodontal model, bordering on the bone tissue of the mandible.

## 3. Results

**Figure 6:**
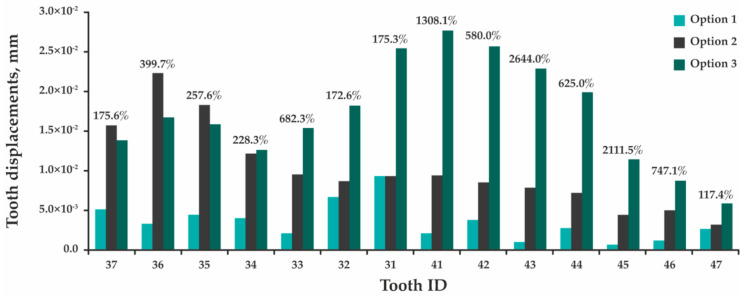
A histogram showing a comparison of the results of determining the maximum total displacements obtained in the models of teeth for options 1, 2, and 3.

**Figure 7:**
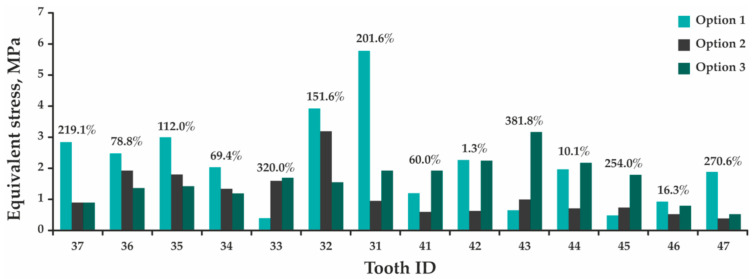
A histogram showing a comparison of the results of determining the maximum equivalent stresses obtained in the models of teeth for options 1, 2, and 3.

**Figure 8:**
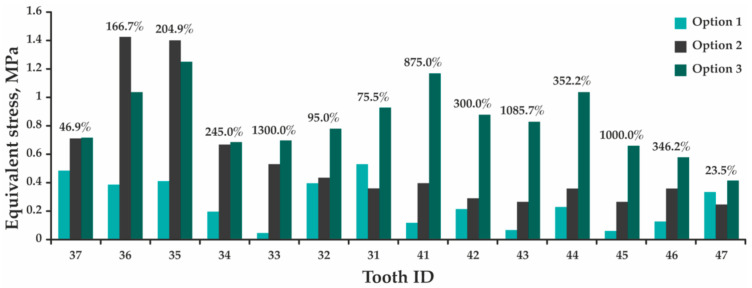
A histogram showing a comparison of the results of determining the maximum equivalent stresses obtained in the models of the periodontal teeth for options 1, 2, and 3.

During the analysis, the maximum total displacement of the teeth was assessed, as well as the maximum equivalent stresses arising in the teeth, PDL, and splint (Table S1). Comparison of the design parameters determining results for each tooth is presented as histograms in Figure 6, Figure 7 and Figure 8, where percentages indicate the maximum difference between rated options 1 and 3.

Figure 6 indicates that the presence of the splint leads to a significant increase in total displacement of all teeth. Despite the fact that the process of splint grinding provides a more uniform distribution of occlusal force, the value of which is 1.5 times lower than in the absence of the splint, the maximum displacement of central incisor 31 increases 2.8 times; incisor 41—14.1 times; and molars 36, 37, 46, and 47—by 5.0, 2.8, 8.5, and 2.2 times, respectively. By assessing stresses, a different picture is observed—the maximum equivalent stresses arising in incisor 31 decrease by 3.0; in incisor 41 increase by 1.6 times; and in molars 36, 37, 46, and 47 decrease by 1.7, 3.2, 1.2, and 3.7 times (Figure 7). In this case, these stresses are concentrated in either the upper part of the tooth root, or at the border of the splint contact with the tooth crown. In the periodontal model, the maximum equivalent stresses occur in the region of teeth roots’ upper part in all design options, and the pattern of stress redistribution indicates a tendency to their significant increase (Figure 8). In option 1, the highest stress is obtained in the periodontium of tooth 31—0.53 MPa, while in option 3—in the periodontium of tooth 35 (1.25 MPa). The maximum equivalent stresses in the splint in option 2 (1.77 MPa, Figure S1a) occur in the area of tooth 36, which receives the maximum load. In option 3, the maximum stresses are concentrated in the space between teeth 42 and 43 (2.70 MPa, Figure S1b) and do not exceed the minimum tensile fracture stress of 32.00 MPa [33].

**Figure 9:**
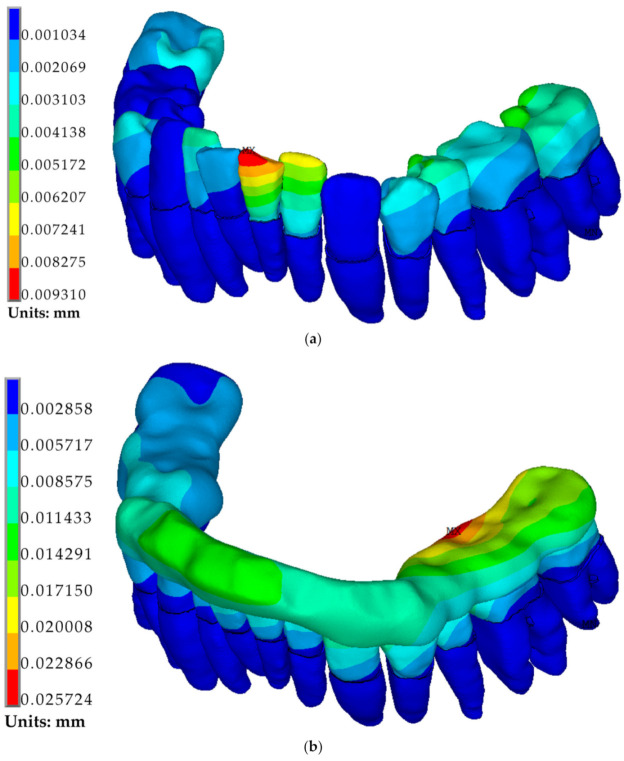

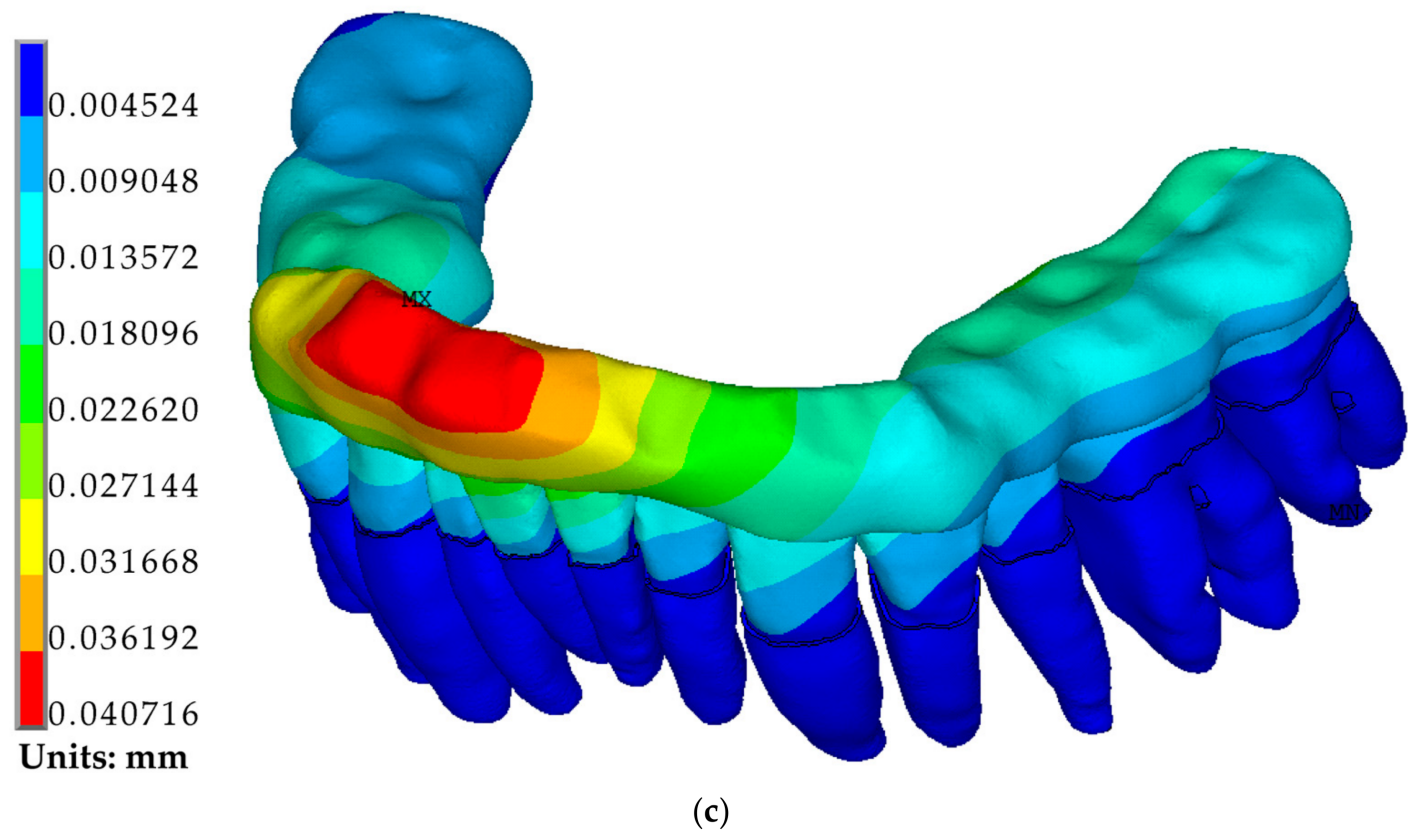
Fields of total displacements distribution in the dentition model: (**a**) for option 1; (**b**) for option 2; and (**c**) for option 3.

From the presented data, it can be seen that in the absence of a splint (Option1), the maximum total displacements occur in the area of the incisor edge of tooth 31 (Figure 9), and the maximum equivalent stresses are in the upper part of the root of the same tooth. Installing a splint obtained after manufacturing (option 2) leads to a two-fold decrease in the occlusal force value and, accordingly, a decrease in the overall level of displacement and stress on all teeth. However, the uneven distribution of this force causes an increased level of stresses and displacements in the teeth of the mandible left side and a lower level in the teeth of the right side. In this case, the maximum displacements in the model are concentrated in the area of tooth 36, which receives the maximum load, and the maximum stresses are in tooth 32, at the border of the splint contact with the tooth crown.

## 4. Discussion

In our previous studies [34,35], where splinted teeth were considered, the similar loading conditions were simulated (a load of 50.0 N acted on each tooth). The use of the T-scan III software and hardware complex was associated with the necessity for a more accurate assessment of the occlusal force level, considering the absence and presence of a splint. Based on the results of experimental studies described in [32], linear relationships were established between the value of the relative occlusal force obtained with the T-scan III and the absolute occlusal force. For this purpose, various jaw prostheses, construction materials simulating the soft tissues of the jaw, and equipment creating a given level of load were used. Since Equation 1 described the experimental data in the best possible way, it was used to determine the absolute occlusal force.

The absolute values of occlusal forces determining results were distributed as follows: maximum value (257.4 N)—in the case of splint absence (measurement 1); minimum value (126.6 N)—in the case of the splint presence installed after manufacture (measurement 2); and medium value (171.8 N)—in the case of splint presence installed after correction. The presence of any splinting device in the oral cavity led to a change in the myotatic effect, i.e., a decrease in the strength of muscle compression (in comparison with occlusion on natural teeth—where a maximum degree of compression exists) and the inability to coordinate one’s muscle efforts in the presence of an insufficient number and uneven distribution of occlusal contacts (occlusal splint immediately after manufacture). The number of occlusal contacts and their distribution uniformity increased after the correction. Such revisions led to occlusal force increase (splint after correction). However, the absence of a signal from the teeth periodontal proprioceptors located under the occlusal splint did not provide the development of the maximum “pathological” load bruxism characteristic (without an occlusal splint). Thus, obtained measurement results were expected.

In accordance with the picture of the first measurement (excluding the splint), the occlusal force was distributed among all teeth with a predominance on the left side of the mandible, and more than half of the entire load fell on molars 36, 37, 46, and 47. One of the reasons for the uneven distribution of occlusal force might be the unevenness of the patient dentition. At the same time, by observing a patient with an even dentition and neuromuscular pathology (bruxism), we could see both uniform and uneven distribution of occlusal force between the teeth. Everything depended on how the chewing muscles work—synchronously or asynchronously.

Despite the fact that the splint was designed by taking into account the processing of CT of the patient’s jaw and the use of modern software, and the manufacture was carried out using digital additive technology, it was not possible to obtain a finished product without subsequent mechanical revision. In the second measurement (using a splint obtained after manufacture), the distribution of the occlusal force remained the same, and 35.0% of the load was taken by molar 36. Additional grinding of the splint made it possible to evenly redistribute the load between the sides of the mandible and the teeth, and the resulting occlusal force decreased 1.5 times compared with measurement 1. The obtained result confirmed the quality of the final splint design, which promoted relaxation of the jaw muscles during occlusion. Changes in the distribution and the overall value of the functional load led to both the achievement of the required therapeutic effect and a decrease in the load on individual parts of the dentition and periodontal tissue, without provoking complications.

Considering the accepted method of fixing the model and the absence of contact interaction between the teeth, in the design option 1, each tooth was an independent model. Since the load was applied in the direction of the contraction vector of the muscles lifting the mandible, and not in the longitudinal axis of the teeth, the process of tooth compression and bending was modeled. In this regard, the maximum stresses arise in the zone of the beginning of the fixation of the teeth (the upper part of the root). In the model of the design options 2 and 3, all the teeth were interconnected through the splint, causing a response to the applied force in each of them. Here, the maximum stresses are also concentrated in the upper part of the roots of the teeth and at the boundaries of the contact of the splint with the crown of the tooth, which is primarily due to the point adherence of the contact surface of the splint model to the crowns of the teeth, causing an uneven distribution of the applied force. Here, the maximum stresses were also concentrated in the upper part of the teeth roots and at the boundaries of the contact of the splint with the crown of the tooth that was primarily due to the point adherence of the splint model contact surface to the teeth crowns causing an uneven distribution of the applied force. In addition, due to the splint construction, the teeth began to perceive an additional bending moment (especially at the anterior part), and their displacement significantly increased (the teeth are additionally displaced anteriorly and downward). Therefore, the maximum displacements obtained in the designs option 2 and 3 are greater than in the design option 1 (see Figure 6). Despite this, in all design options, the values of the maximum total displacements are within the physiological mobility of healthy teeth for a given type of loading (vertical direction of the load action)—up to 0.03 mm [36], and the obtained maximum equivalent stresses are significantly lower than the ultimate strength of dentin: ultimate tensile strength—from 44.40 to 97.80 MPa [37], ultimate compressive strength—297.20 MPa [38]. The maximum stresses arising in the PDL are also below the allowable value for this type of tissue and the age group (20–49 years): incisors—1.50–1.60 MPa, canines—1.60–1.70 MPa, premolars—1.40 MPa, and molars—1.20 MPa [39]. This allows us to conclude that the installation of the splint does not lead to any damage to the teeth and surrounding tissues under the considered effect of the functional load.

The level of the maximum equivalent stress occurring in the splint is relatively low. Obviously, a single loading of a given type does not lead to a breakdown of the presented design splint, while the assessment of the cyclic strength can be of decisive importance when choosing its design parameters of the splint and material performance. Thus, in the study [40], the analysis of the fatigue life of PMMA was carried out, and the corresponding fatigue curves are presented depending on the frequency of loading of the samples and the damage accumulation model. Using these models, it can be determined that for the obtained stress level (maximum equivalent stress arising in the splint—2.70 MPa), the number of loading cycles before breakage would be more than 10 million. If we assume that with parafunction, the loading conditions correspond to the conditions considered above, then the splint would retain its functionality for a long period of operation (up to 6 months). However, in the process of eccentric movements, local areas of the splint would perceive an increased level of load; the direction of which is not constant. Therefore, for a more reliable assessment of the splint service life, it is necessary to conduct additional studies, taking into account all possible conditions of its loading during the operation of the mandible.

It should be noted that this study considered the option of using a splint on the mandible. However, the treatment of bruxism can be successfully implemented by placing a splint on the maxilla. Such a splint is distinguished by design and has a number of advantages. At the moment, we are conducting a study for maxillary splints, where the same study protocol is applied.

## 5. Limitation of the Study

In this study, by creating a finite element model of the mandible, only teeth and periodontium were considered, and consideration of the mandible bone tissue was not included in the research objectives. This is primarily due to the need to reduce the time to create a model and its dimensions. The bone tissue of the mandible contains a volume that exceeds the total volume of the teeth and periodontium; therefore, while maintaining the settings for the approximation of the model (the maximum length of the side of the tetrahedron face is 0.70 mm), the total of the elements and nodes of the model increases significantly, placing increased requirements on the calculated powers or increasing the time performing the calculation. Thus, the model did not take into account the flexibility of the mandible bone tissue, reducing the accuracy of determining the stress–strain state of the model components.

In addition, we assumed that the fit of the splint inner surface to the dental crowns is ideal (without gaps and interference), and we did not consider the possibility of moving the splint relative to the tooth surface. Therefore, the simplest contact interaction (bonded) between the splint and the teeth was chosen.

However, in our opinion, the use of such an approach does not affect the general conclusions of the study.

The empirical results presented here should be considered with caution because of the lack of probability sampling. The absence of the statistically acceptable subject of the sample in the study is associated with the use of a new methodology for assessing the effectiveness of treatment.

The results obtained by us are valid for the stated particular case; however, a representative sample is required, which should be taken into account in further research in this direction.

## 6. Conclusions

Based on the results of the study, the following conclusions can be drawn:

- The presented technology makes it possible to take into account the individual geometric characteristics of the jaw components (teeth, periodontium, and dentition, in general) of a particular patient when planning dental orthopedic treatment of mastication muscles parafunction.
- The results of the study proved the effectiveness of using the splint in order to change the distribution of functional load in the treatment of patients with mastication muscles parafunction.
- The potential applicability of PMMA as a splint material for the treatment of mastication muscle parafunction has been established. However, additional research is required, taking into account the various loading conditions that arise during the operation of the jaw (biting processes and unilateral chewing).

## Figures and Tables

**Table 1:** Mechanical characteristics of materials.

Material Name	Young’s Modulus (MPa)	Poisson’s Ratio
Tooth [30]	20,300.00	0.26
PDL [31]	68.90	0.45
PMMA [28,29]	2200.00	0.35

**Table 2:** Distribution of absolute values obtained as a result of data processing of the software and hardware complex T-Scan III.

Tooth ID	Measurement 1 [N (%)]	Measurement 2 [N (%)]	Measurement 3 [N (%)]
37	43.8 (17)	6.3 (5)	13.7 (8)
36	54.0 (21)	44.3 (35)	24.1 (14)
35	18.0 (7)	16.4 (13)	17.2 (10)
34	10.3 (4)	11.4 (9)	8.6 (5)
33	2.6 (1)	6.3 (5)	8.6 (5)
32	15.4 (6)	1.3 (1)	6.9 (4)
31	15.4 (6)	1.3 (1)	6.9 (4)
41	2.6 (1)	1.3 (1)	3.4 (2)
42	7.7 (3)	5.1 (4)	20.6 (12)
43	5.1 (2)	5.1 (4)	12.0 (7)
44	10.3 (4)	3.8 (3)	10.3 (6)
45	5.1 (2)	2.5 (2)	13.7 (8)
46	20.6 (8)	15.2 (12)	15.5 (9)
47	46.3 (18)	6.3 (5)	10.3 (6)

N, Newton; %, percentage.

## Data Availability

The data presented in this study are available on request from the corresponding author.

## Supplementary Materials

The following are available online at https://www.mdpi.com/article/10.3390/jfb12020023/s1, Table S1: results of determining the stress-strain state of the model components, Table S2: results of determining the equivalent stresses for the components of the mandible model, Figure S1: fields of the equivalent stress distribution in the splint model: (**a**) for option 2; (**b**) for option 3, Figure S2: occlusal view of the patient’s: (**a**) upper arch; (**b**) lower arch., Figure S3: occlusal view of the virtual model of the patient’s: (**a**) upper arch; (**b**) lower arch, Figure S4: frontal view of the virtual model of the patient’s: (**a**) upper arch; (**b**) lower arch, Figure S5: process of occlusion measuring by T-Scan III with physical model of the splint (after grinding correction): (**а**) view from above; (**b**) bottom view.
