# Supplementary material for: Personalized Biomechanical Analysis of the Mandible Teeth Behavior in the Treatment of Masticatory Muscles Parafunction
Source: J Funct Biomater. 2021 Apr 9;12(2):23. doi: 10.3390/jfb12020023 (PMC8167636; doi:10.3390/jfb12020023)
Supplement: Supplementary file 1 [file jfb-12-00023-s001.pdf]

Table S1. Results of determining the stress-strain state of the model components.

Tooth ID	Option 1			Option 2			Option 3		
	Maximum displacement (mm)	Maximum von Mises Stress (MPa)		Maximum displacement (mm)	Maximum von Mises Stress (MPa)		Maximum displacement (mm)	Maximum von Mises Stress (MPa)	
		T	PDL		T	PDL		T	PDL
37	$5.05 \cdot \times 10^{-3}$	2.84	0.49	$15.85 \cdot \times 10^{-3}$	0.91	0.71	$13.92 \cdot \times 10^{-3}$	0.89	0.72
36	$3.36 \cdot \times 10^{-3}$	2.45	0.39	$22.47 \cdot \times 10^{-3}$	1.91	1.43	$16.79 \cdot \times 10^{-3}$	1.37	1.04
35	$4.46 \cdot \times 10^{-3}$	3.01	0.41	$18.31 \cdot \times 10^{-3}$	1.79	1.40	$15.95 \cdot \times 10^{-3}$	1.42	1.25
34	$3.82 \cdot \times 10^{-3}$	2.05	0.20	$12.16 \cdot \times 10^{-3}$	1.33	0.67	$12.54 \cdot \times 10^{-3}$	1.21	0.69
33	$1.98 \cdot \times 10^{-3}$	0.40	0.05	$9.59 \cdot \times 10^{-3}$	1.60	0.53	$15.49 \cdot \times 10^{-3}$	1.68	0.70
32	$6.75 \cdot \times 10^{-3}$	3.95	0.40	$8.72 \cdot \times 10^{-3}$	3.19	0.44	$18.40 \cdot \times 10^{-3}$	1.57	0.78
31	$9.31 \cdot \times 10^{-3}$	5.82	0.53	$9.34 \cdot \times 10^{-3}$	0.94	0.36	$25.63 \cdot \times 10^{-3}$	1.93	0.93
41	$1.98 \cdot \times 10^{-3}$	1.20	0.12	$9.39 \cdot \times 10^{-3}$	0.60	0.40	$27.88 \cdot \times 10^{-3}$	1.92	1.17
42	$3.80 \cdot \times 10^{-3}$	2.26	0.22	$8.60 \cdot \times 10^{-3}$	0.62	0.29	$25.84 \cdot \times 10^{-3}$	2.23	0.88
43	$0.84 \cdot \times 10^{-3}$	0.66	0.07	$7.83 \cdot \times 10^{-3}$	0.97	0.27	$23.05 \cdot \times 10^{-3}$	3.18	0.83
44	$2.76 \cdot \times 10^{-3}$	1.98	0.23	$7.16 \cdot \times 10^{-3}$	0.72	0.36	$20.01 \cdot \times 10^{-3}$	2.18	1.04
45	$0.52 \cdot \times 10^{-3}$	0.50	0.06	$4.47 \cdot \times 10^{-3}$	0.74	0.27	$11.50 \cdot \times 10^{-3}$	1.77	0.66
46	$1.04 \cdot \times 10^{-3}$	0.93	0.13	$4.98 \cdot \times 10^{-3}$	0.52	0.36	$8.81 \cdot \times 10^{-3}$	0.80	0.58
47	$2.70 \cdot \times 10^{-3}$	1.89	0.34	$3.26 \cdot \times 10^{-3}$	0.38	0.25	$5.87 \cdot \times 10^{-3}$	0.51	0.42

T, tooth; PDL, periodontal ligament.

Table S2. Results of determining the equivalent stresses for the components of the mandible model.

<table>
<tr><th>Component</th><th>Option 1</th><th>Option 2</th><th>Option 3</th></tr>
<tr><td>Tooth 31</td><td>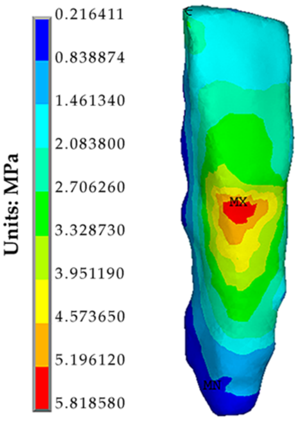</td><td>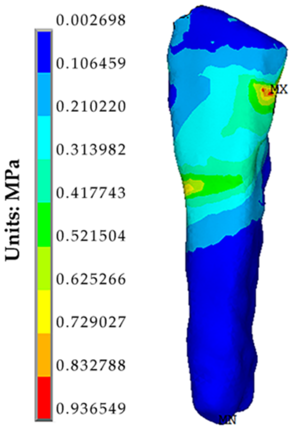</td><td>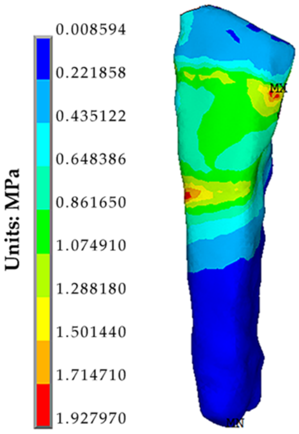</td></tr>
<tr><td>PDL of tooth 31</td><td>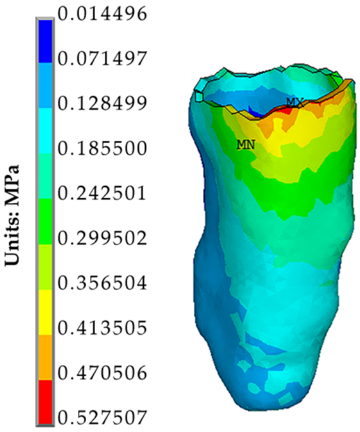</td><td>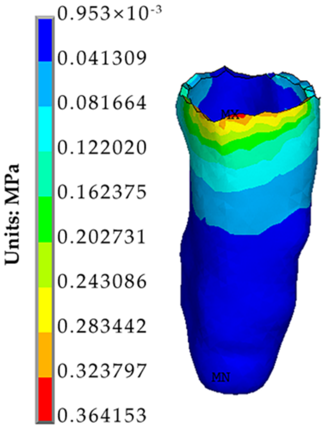</td><td>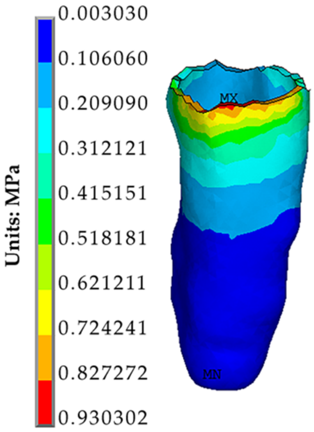</td></tr>
<tr><td>Tooth 37</td><td>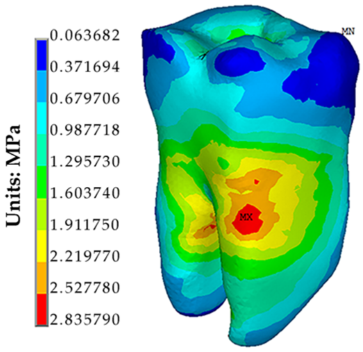</td><td>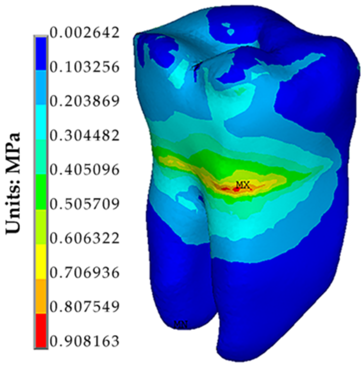</td><td>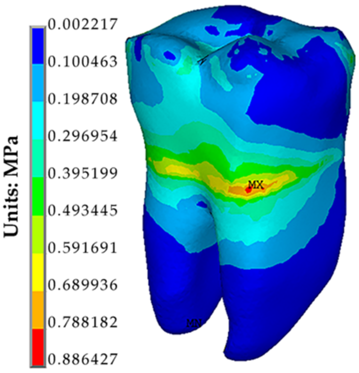</td></tr>
<tr><td>PDL of tooth 37</td><td>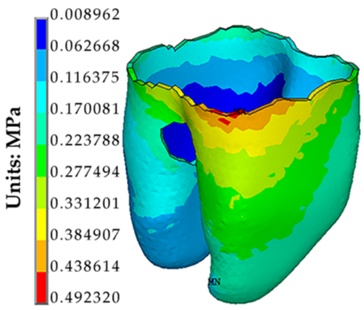</td><td>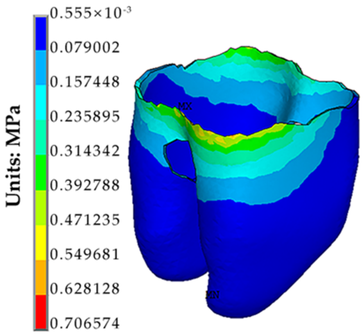</td><td>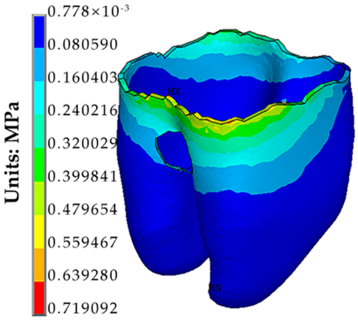</td></tr>
</table>

PDL, periodontal ligament.

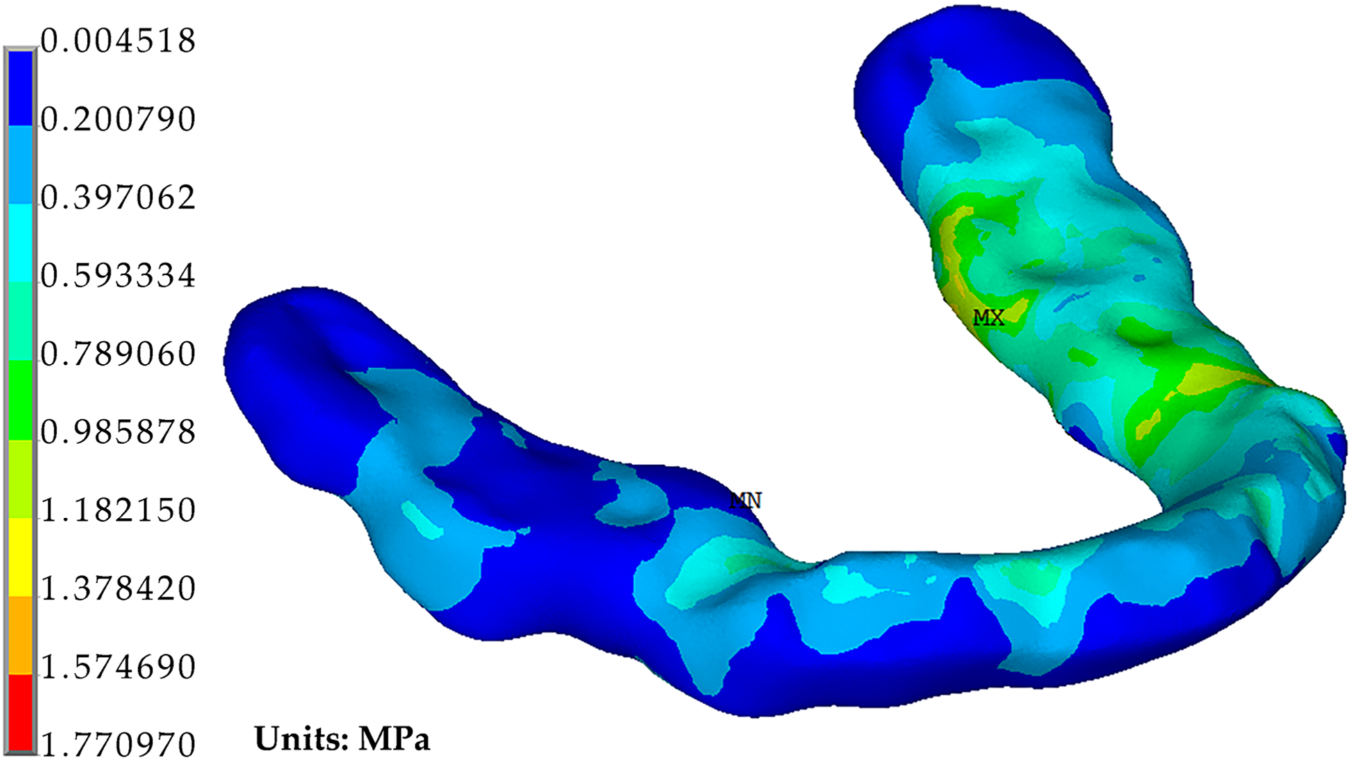

(a)

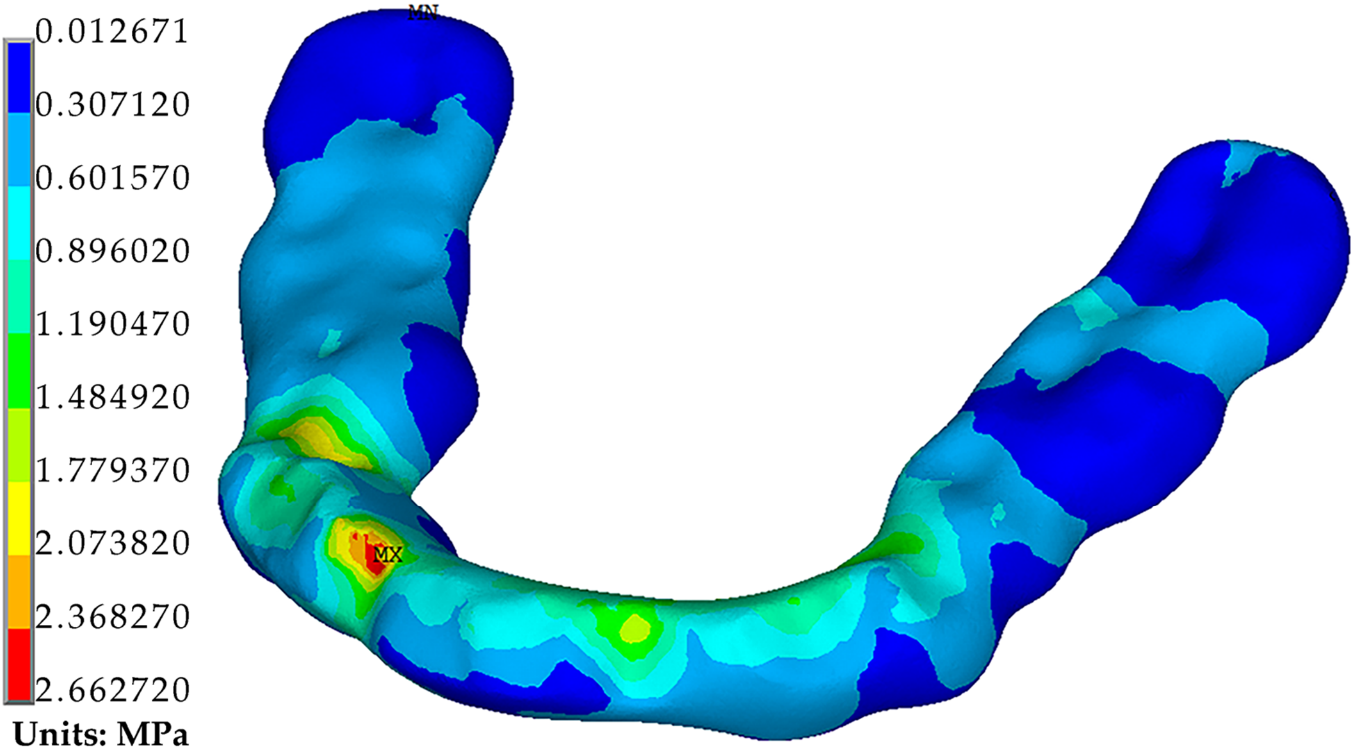

(b)

Figure S1. Fields of the equivalent stress distribution in the splint model: (**a**) for Option 2; (**b**) for Option 3.

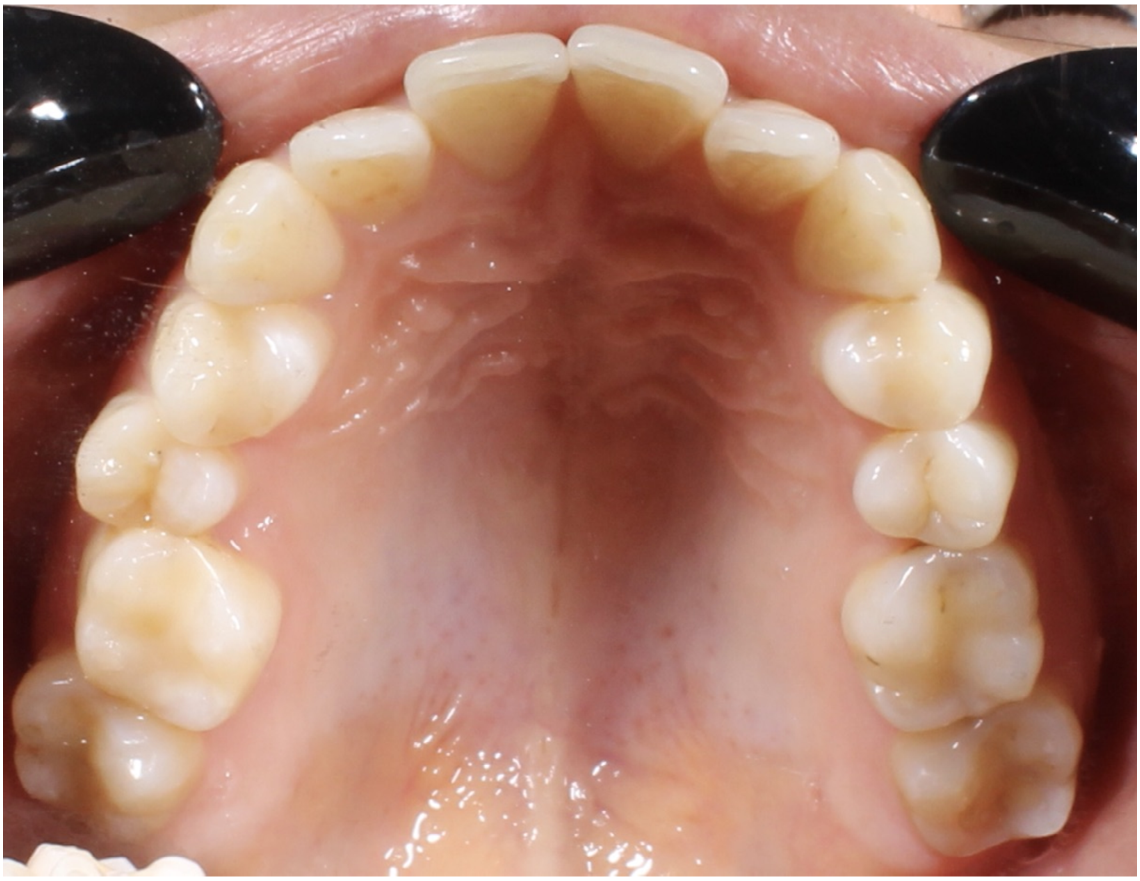

(a)

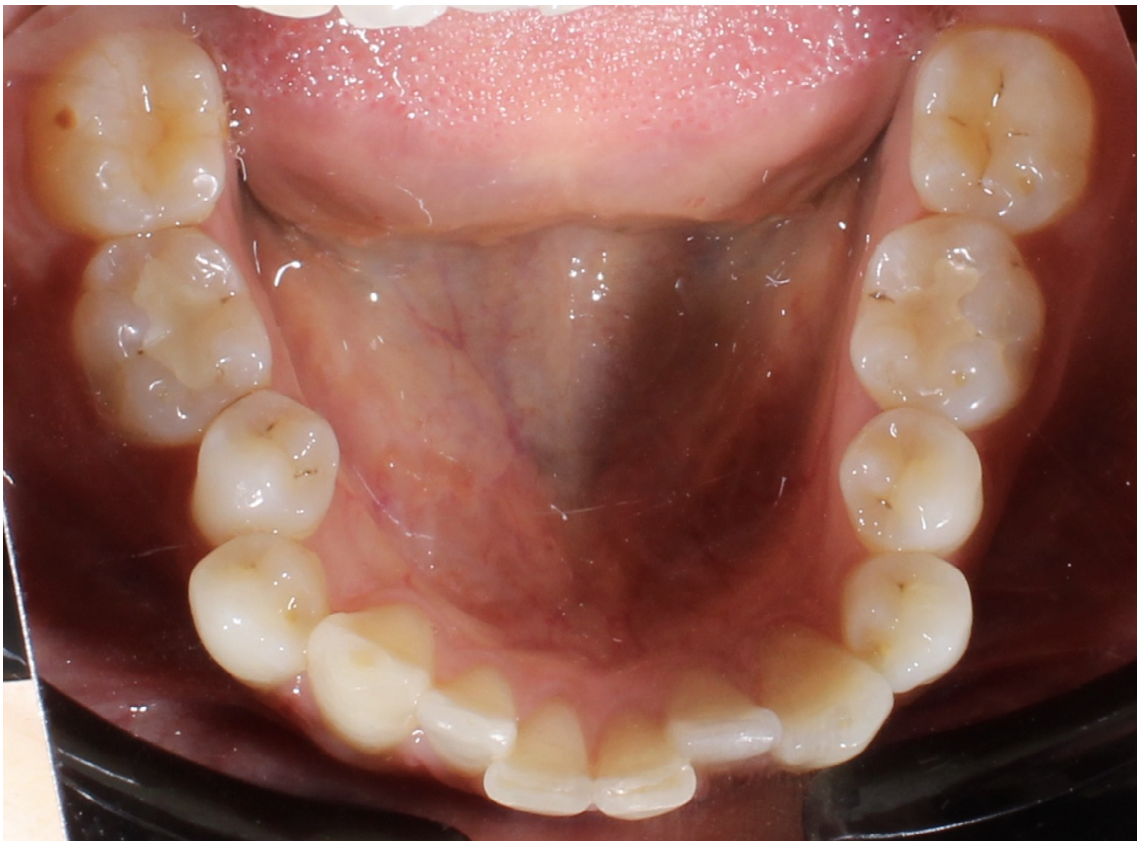

(b)

Figure S2. Occlusal view of the patient's: (**a**) upper arch; (**b**) lower arch.

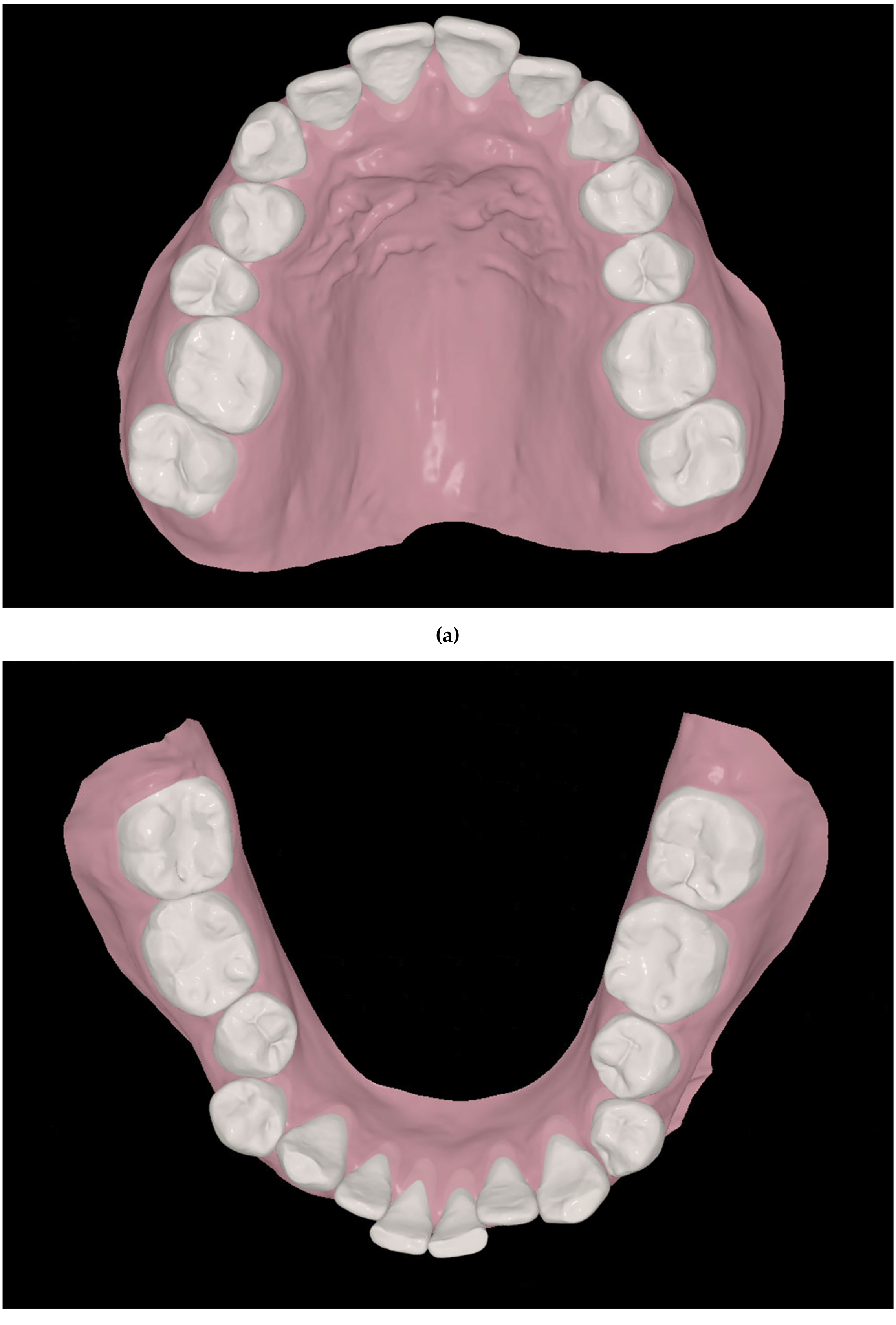

(a)

(b)

Figure S3. Occlusal view of the virtual model of the patient's: (**a**) upper arch; (**b**) lower arch.

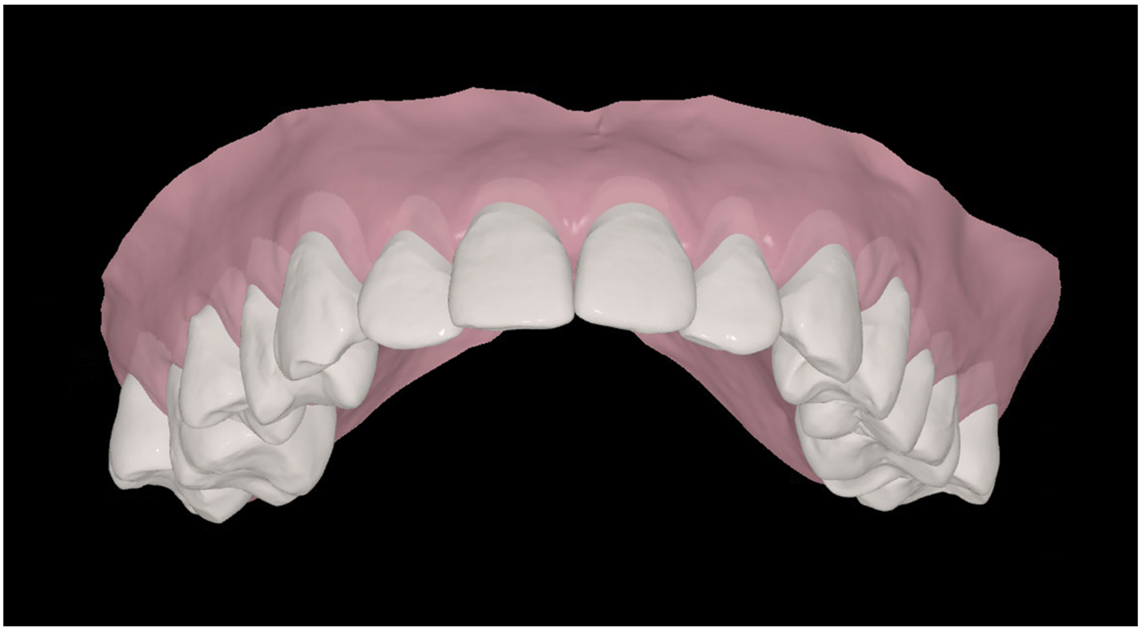

(a)

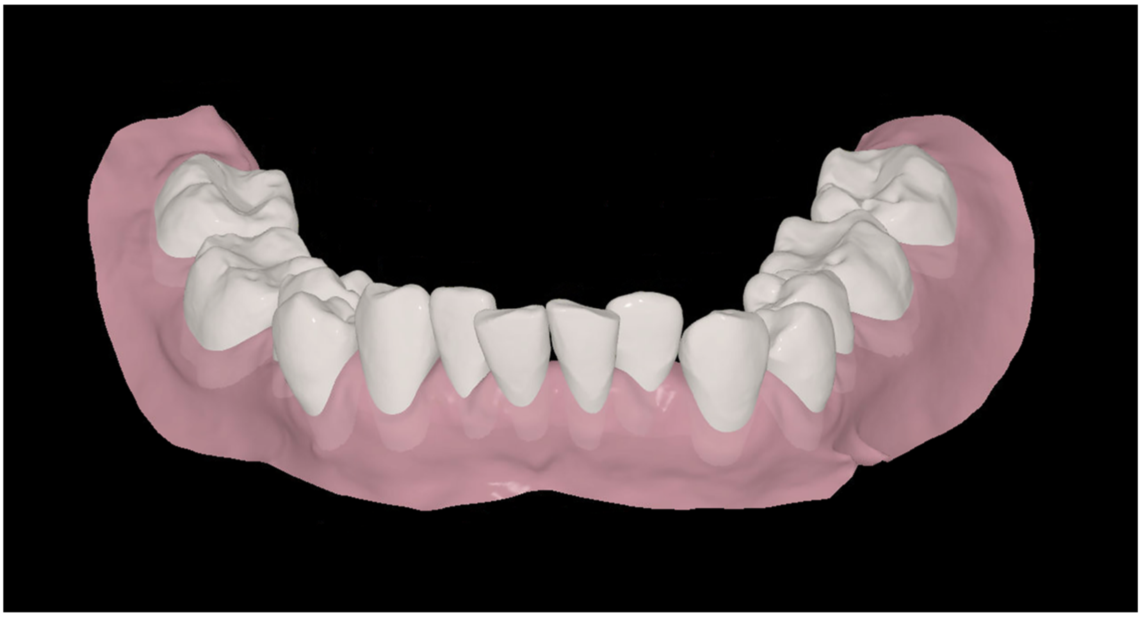

(b)

Figure S4. Frontal view of the virtual model of the patient's: (**a**) upper arch; (**b**) lower arch.

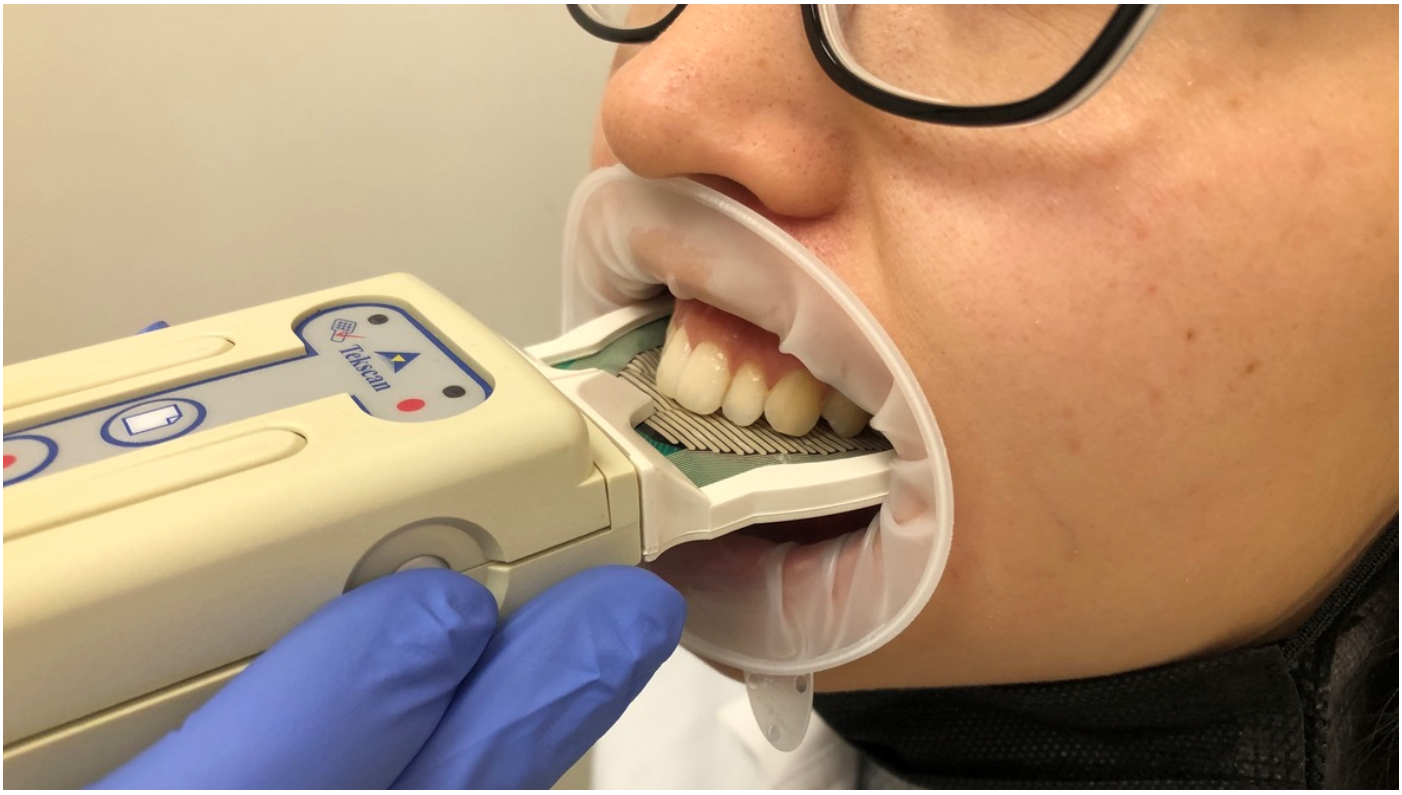

(a)

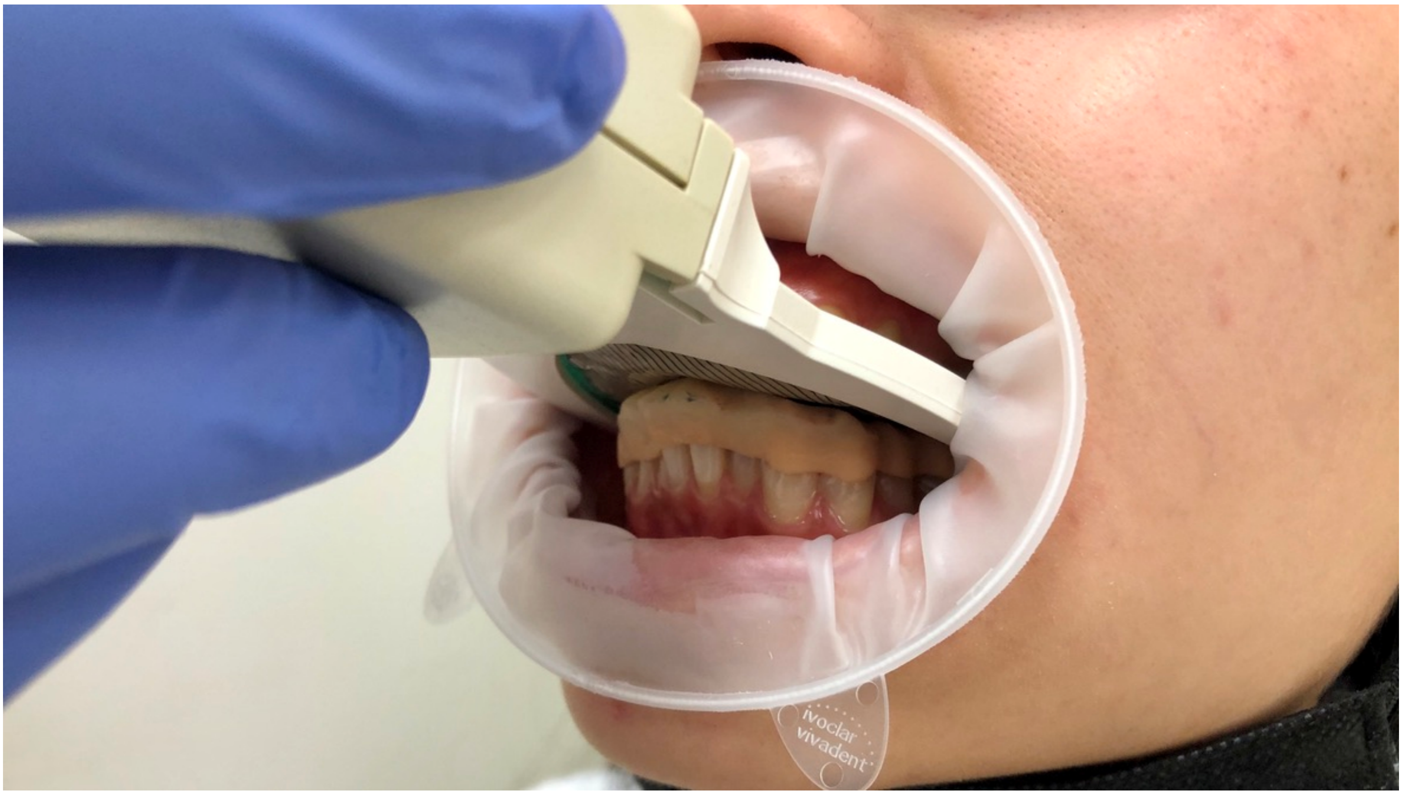

(b)

Figure S5. Process of occlusion measuring by T-Scan III with physical model of the splint (after grinding correction): (**a**) view from above; (**b**) bottom view.
